# Enhancing physiologic simulations using supervised learning on coarse mesh solutions

**DOI:** 10.1098/rsif.2014.1073

**Published:** 2015-03-06

**Authors:** Kumaran Kolandaivelu, Caroline C. O'Brien, Tarek Shazly, Elazer R. Edelman, Vijaya B. Kolachalama

**Affiliations:** 1 Institute for Medical Engineering and Science, Massachusetts Institute of Technology, Cambridge, MA 02139, USA; 2 David H. Koch Institute for Integrative Cancer Research, Massachusetts Institute of Technology, Cambridge, MA 02139, USA; 3 Cardiovascular Division, Brigham and Women's Hospital, 75 Francis Street, Boston, MA 02115, USA; 4 College of Engineering and Computing, University of South Carolina, Columbia, SC 29208, USA; 5 Charles Stark Draper Laboratory, 555 Technology Square, Cambridge, MA 02139, USA

**Keywords:** computational modelling, machine learning, Gaussian process, nearest neighbours, drug-coated balloons, drug-eluting stents

## Abstract

Computational modelling of physical and biochemical processes has emerged as a means of evaluating medical devices, offering new insights that explain current performance, inform future designs and even enable personalized use. Yet resource limitations force one to compromise with reduced order computational models and idealized assumptions that yield either qualitative descriptions or approximate, quantitative solutions to problems of interest. Considering endovascular drug delivery as an exemplary scenario, we used a supervised machine learning framework to process data generated from low fidelity coarse meshes and predict high fidelity solutions on refined mesh configurations. We considered two models simulating drug delivery to the arterial wall: (i) two-dimensional drug-coated balloons and (ii) three-dimensional drug-eluting stents. Simulations were performed on computational mesh configurations of increasing density. Supervised learners based on Gaussian process modelling were constructed from combinations of coarse mesh setting solutions of drug concentrations and nearest neighbourhood distance information as inputs, and higher fidelity mesh solutions as outputs. These learners were then used as computationally inexpensive surrogates to extend predictions using low fidelity information to higher levels of mesh refinement. The cross-validated, supervised learner-based predictions improved fidelity as compared with computational simulations performed at coarse level meshes—a result consistent across all outputs and computational models considered. Supervised learning on coarse mesh solutions can augment traditional physics-based modelling of complex physiologic phenomena. By obtaining efficient solutions at a fraction of the computational cost, this framework has the potential to transform how modelling approaches can be applied in the evaluation of medical technologies and their real-time administration in an increasingly personalized fashion.

## 1. Introduction

Emerging applications in clinical medicine and medical device design are just beginning to embrace the potential of computational modelling [1,2]. In the face of increasing interest by medical device companies, regulatory agencies [3] and clinicians [4,5], it is critical that we find approaches that yield accurate information quickly—whether it is in the optimization of device design over a wide parameter space [6–9], to better understand device and implant performance in patient-specific environments [10–16], or even to perform virtual feasibility studies for procedural planning (i.e. implantation of stents, endovascular grafts, heart valves, bioprosthetics, etc.) [17–20]. Particularly in clinical applications, speed and efficiency are paramount as results must be provided cost-effectively and often in urgent settings (i.e. within minutes). Historically, efficiency and practicality have trumped accuracy due to resource limitations associated with computational hardware and software as well as access to relevant information. The last decades have witnessed a remarkable explosion of medical knowledge (both multi-scale and multi-physics) as well as growth in our ability to access and store data in real-time, patient-specific formats [21]. Yet processing this knowledge has outstripped the exponential growth in high-performance computing and even the most promising, clinically relevant applications to date remain fundamentally limited by computational delays [22]. To fully leverage the possibilities promised by simulation, it is critical that we find methods that better handle information without loss of efficiency or accuracy.

Cardiovascular applications are among the most important and widely studied cases of medical simulation. Devices such as drug-eluting stents [23,24] and balloon catheters [25–28] are used millions of times each year to help manage coronary heart disease, the leading cause of global mortality [29]. In this disease, blockages build up in the blood vessels supplying the heart and must be reopened to prevent untoward cardiovascular events. Both disease progression as well as the success and failure of such devices depend on local physical and biochemical processes coupled within complex physiological environments. While local delivery of drugs helps modulate device response, the drugs themselves are highly toxic (often chemotherapeutics) characterized by a narrow therapeutic window—delivery of too little compromises effectiveness; delivery of too much compromises safety [30,31]. Designing and achieving optimal device performance requires that we understand the confluence of fluid flow, drug transport and tissue reactivity coupled within relevant, often patient-specific environments.

**Figure 1.**
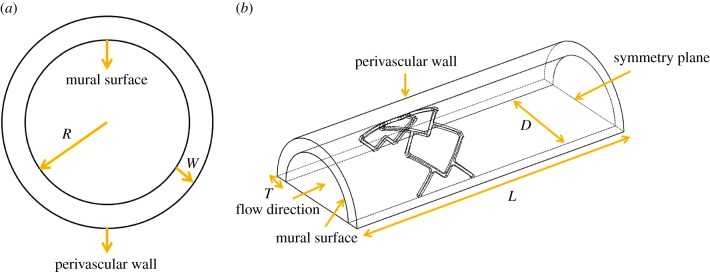
Schematics of the two computational models used for the studies. (*a*) A two-dimensional model of an expanded drug-coated balloon is shown that transfers drug to the tissue-lumen (or mural) surface. *R* = 3 mm indicates the lumen radius and *W* = 0.5 mm is the arterial wall thickness. (*b*) A three-dimensional model of a single cell, delta-winged stent deployed in an arterial vessel is shown with drug releasing from the stent surface. Here, the lumen diameter was defined as *D* = 3.6 mm, arterial wall thickness as *T* = 0.35 mm, domain length as *L* = 11.36 mm. The upstream section length relative to the stent position was defined as 3 mm and the downstream section length as 6 mm. (Online version in colour.)

We sought to determine whether supervised machine learning can be leveraged to estimate higher fidelity data from lower fidelity computational datasets, considering two computational mesh-based simulations of distinct computational complexities. The first is a two-dimensional time-dependent model of drug release, transport and reversible tissue binding from a drug-coated balloon (figure 1*a*); the second a steady-state simulation of blood flow coupled with species transport and reversible tissue binding in a three-dimensional model of a drug-eluting stent deployed in an arterial vessel (figure 1*b*). Using Gaussian process modelling (GPM) [32], we constructed supervised learners that used lower resolution mesh results to predict solutions of higher mesh density. Computational simulations were performed on a low fidelity, mesh configuration (baseline) comprised of variably sized elements that captured underlying physics with reasonable accuracy as well as on mesh configurations of increasing density (Refinements 1, 2 and 3). Nearest neighbour information of a reference mesh node for a low fidelity configuration (baseline) along with low fidelity solutions at these nearest neighbours and at the reference mesh node defined the input feature vector for the supervised learner. With the true solution of the reference mesh node at the intermediate mesh setting (Refinement 1) serving as scalar output, GPM was used to construct computationally inexpensive surrogates to predict higher fidelity solutions (Refinement 2); these predictions were cross-validated using the true solutions obtained from the highest mesh density (Refinement 3). Our results demonstrate the potential of applying supervised machine learning techniques to approximate physics-based solutions both cost-effectively and accurately, providing a paradigm for emerging applications in medicine.

## 2. Material and methods

### 2.1. Model of drug-coated balloon therapy

Drug-coated balloons have recently emerged as viable therapeutic options for treating obstructive arterial disease [25–28]. With this technology, short-term transfer of therapeutic drugs to the arterial wall can be achieved without the requirement of permanent indwelling delivery systems. Transient arterial tissue distribution from an expanded drug-coated balloon was modelled as a two-dimensional, time-dependent continuum transport problem. The computational domain constituted an arterial cross-section with radius (*R* = 3 mm) and wall thickness (*W* = 0.5 mm) (figure 1*a*). Free drug was allowed to diffuse with a constant diffusivity (*D*_w_) and reversibly bind to tissue sites according to the reaction–diffusion equation2.1
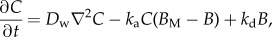
where *C* and *B* denote the local concentrations of free and bound drug in the arterial wall, respectively. *D*_w_ = 1.712 × 10^−11^ m^2^ s^−1^ is the apparent net diffusivity, *B*_M_ = 0.356 mmol l^−1^ is the net tissue binding capacity [33], and *k*_a_ and *k*_d_ are the association and dissociation rate constants, respectively, for the model drug (zotarolimus). *k*_a_ and *k*_d_ were computed as *k*_a_ = *D*_w_*D*_a_/*B*_M_*W*^2^ and *k*_d_ = *k*_a_*k*_d_, where *Da* = 50 000 is the Damköhler number and *k*_d_ = 0.0326 mmol l^−1^ is the equilibrium dissociation constant [33]. Equation (2.1) was solved subject to zero initial free and bound drug concentrations within the tissue, a perfect sink condition at the adventitial surface and a flux boundary condition at the mural surface defined as2.2
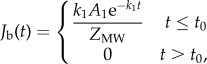
where *J*_b_(*t*) is the flux approximating the releasable portion of zotarolimus from the balloon during inflation, *t*_0_ = 30 s is the balloon inflation time, *A*_1_ = 23.95 kg m^−3^ and *k*_1_ = 0.009208 s^−1^ are empirical constants estimated from bench-top release kinetics experiments [33], and *Z*_MW_ = 966.21 g mol^−1^ is the molecular weight of zotarolimus. A zero concentration condition was applied on the perivascular side of the arterial wall for the free drug. For the bound drug, both the lumen-tissue (or mural) and the perivascular aspects of the arterial wall were assigned a zero flux boundary condition. Time-dependent simulations (COMSOL 4.3a, Comsol Inc.) were performed on the computational domain that was meshed using the Delaunay triangulation scheme. Three model configurations were then constructed by sequentially refining the entire domain using longest edge refinement technique, where the longest edge of each mesh element is bisected at each of the three levels, defined as Refinements 1, 2 and 3 (true solution), respectively (table 1*a*). The Direct (SPOOLES) method was used to solve the system of equations with a nested dissection pre-ordering algorithm and a backward differentiation formula method was used for time stepping with relative and scaled absolute tolerances assigned at 1 × 10^−9^ and 1 × 10^−6^, respectively. Simulations were performed until the minimum damping and tolerance factors reached 1 × 10^−9^ and 1 × 10^−6^, respectively. Arterial tissue distributions of free and bound drug for three model configurations of increasing mesh density were extracted at 1 h from balloon inflation for supervised learning.

**Table 1.** Mesh configurations of increasing density for (*a*) two-dimensional time-dependent model simulating drug-coated balloon therapy and (*b*) three-dimensional steady-state model of drug-eluting stent therapy. Mesh refinement was performed by bisecting the longest edge of each element. The increase in computational time (rounded to the tenths place) of the true solution scales with mesh density. Simulations were performed on a dual-core Intel Xeon X5687 @ 3.6 GHz processor with an installed memory of 72 GB.

mesh setting	cell count	degrees of freedom	simulation time (hours)
(*a*)			
baseline	5302	5890	0.8
Refinement 1	11 326	12 500	2.5
Refinement 2	23 861	25 042	3.8
Refinement 3	49 829	52 178	5.8
(*b*)			
baseline	83 397	211 981	0.3
Refinement 1	333 445	848 238	2.6
Refinement 2	1 077 508	2 709 490	65.7

### 2.2. Model of stent-based drug therapy

Unlike drug-coated balloons, the metallic drug-eluting stents remain permanently implanted at the lesion site but can provide sustained release of therapeutic drugs. Owing to their remarkable clinical success, they are considered as the primary choice for treating coronary artery disease [23,24]. A three-dimensional computational model of a drug-eluting stent deployed in a non-bifurcating arterial vessel was constructed (SolidWorks, Dassault Systèmes) [34,35]. The diameter of the arterial vessel was defined as *D* = 3.6 mm, arterial wall thickness as *T* = 0.35 mm and vessel length as *L* = 11.36 mm. A fully apposed single cell, delta-wing shaped slotted tube design was used for the stent with diameter 3.5 mm, and the intrinsic strut shape modelled as a square with dimensions 10^−4^ × 10^−4^ m^2^. The effects of pulsatile flow were approximated using a steady-state flow equivalent within this artery–stent combination, as this approximation was used to produce a physiologically realistic assessment of the impact of flow on arterial drug distribution [36]. In the arterial lumen, the continuity and momentum equations2.3
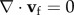
and2.4
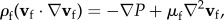
respectively were solved where **v**_f_, *ρ*_f_ = 1060 kg m^−3^, *P* and *μ*_f_ = 3.5 × 10^−3^ Pa · s are, respectively, the velocity, density, pressure and the viscosity of flowing blood. The arterial wall was assumed to be a porous medium where the continuity equation2.5
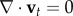
was solved, where ***v****_t_* is the interstitial fluid velocity. The momentum equation2.6
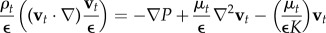
was assumed to follow Darcy's Law, where *K* = 1.43 × 10^−18^ m^2^ is Darcy's wall permeability, *ε* = 0.43 is wall porosity, *ρ*_*t*_ = 1000 kg m^−3^ is fluid density and *μ*_*t*_ = 8.9 × 10^−4^ Pa · s fluid viscosity within the arterial wall [37]. A constant velocity profile was prescribed at the luminal inlet. At the outlet, a zero pressure boundary condition was set. No-slip boundary conditions were imposed on the strut-blood and mural interfaces. The inlet condition fixed at a constant Reynolds number 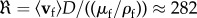 was based on mean blood flow and diameter measurements obtained from the human left anterior descending coronary artery [37].

Drug transport in the lumen was modelled as an advection–diffusion process defined as2.7
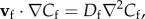
where *C*_f_ denotes lumen drug concentration and *D*_f_ = 3.89 × 10^−11^m^2^ s^−1^ is the model drug (paclitaxel) diffusivity in the lumen [37,38]. Drug transport in the arterial wall follows an advection–diffusion–reaction model as follows:2.8
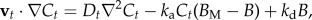
where *D_t_* = 3.65 × 10^−12^ m^2^ s is paclitaxel diffusivity in the arterial wall [37]. *C_t_* and *B* denote the local concentrations of free and bound drug in the arterial wall, respectively, *B*_M_ = 1.3 mM is the net tissue binding capacity [33], and *k*_a_ and *k*_d_ are the association and dissociation rate constants, respectively. Flux continuities for drug transport were maintained at the mural interface. *k*_a_ and *k*_d_ were computed as *k*_a_ = *D_t_Da*/*B*_M_*T*^2^ and *k*_d_ = *k*_a_*K*_d_, where *Da* = 2700 is the Damköhler number and *k*_d_ = 0.136 mM is the equilibrium dissociation constant for paclitaxel [33].

A finite-element solver (COMSOL 4.3a, Comsol Inc.) was used to perform the coupled flow and drug transport simulations. The three-dimensional computational domain was discretized using tetrahedral control volumes with a linear shape function into an initial mesh (baseline). The mesh density was highest near the strut and the mural surface and then decreased towards the centreline of the lumen. Sequential mesh refinement using the longest edge bisection method was then performed to create cases with refined mesh densities (table 1*b*). An open boundary condition for drug concentration was applied at the inlet and the outlet of the arterial lumen. An impermeable boundary condition was established at the perivascular aspects of the model vessel. Stent drug release was simulated using a Dirichlet boundary condition of unit concentration. For the bound drug, the intramural and the perivascular aspects of the arterial wall, tissue inlet and tissue outlet were all assigned a zero flux boundary condition. Symmetry boundary conditions were exploited on the model geometry as the stent location was far from the lumen centreline. The physiologic rates of luminal blood flow coupled with transport of low diffusive drugs generate high Peclet numbers within the computational domain. An upwind Petrov–Galerkin streamline diffusion formulation was therefore enforced with full residual to stabilize the governing equations. Simulations were performed for computational configurations of increasing mesh densities and until there was a 1 × 10^−9^ reduction in the mass transport residuals for each case. The solutions and mesh information for all cases were subsequently processed for supervised learning.

### 2.3. Supervised learning framework

**Figure 2.**
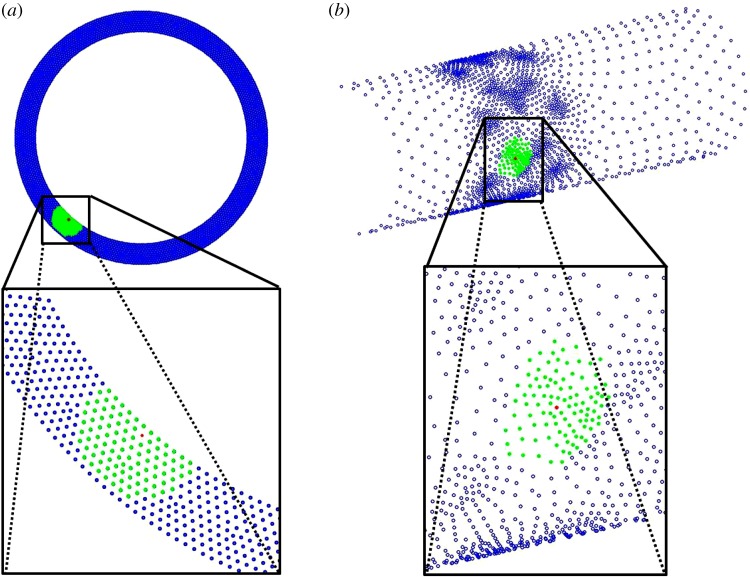
Schematic representing a finite number of nearest neighbours (denoted in green) that were extracted for a specific node (denoted in red) on the computational domains for the model simulating drug-coated balloon therapy (*a*) and the one simulating drug-eluting stent therapy (*b*). Computational mesh nodes were denoted in blue.

Supervised learning is a branch of machine learning where functions are inferred from labelled training data comprising a set of variables called inputs and their corresponding outputs. The outputs can be qualitative/categorical or quantitative in nature, with the corresponding learning problem denoted as ‘classification’ or ‘regression’, respectively [39,40]. In this paper, a construct for the training data defined by an input vector 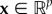 trained with a scalar output *y*(**x**) was used for supervised learning. Numerical solutions on the mesh configurations of increasing density followed by computation of the pairwise Euclidean distances for each node's nearest neighbours (figure 2*a*,*b*) within each computational mesh configuration were obtained [41]. During model training, each node (*x_i_*) and the nearest neighbour (*x_j_*) have a pairwise distance2.9
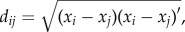
for the baseline mesh configuration. Note that 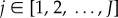, where *J* denotes the number of nearest neighbours. These pairwise distances along with the corresponding output for the baseline mesh configuration2.10
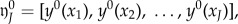
were included as part of the input feature space 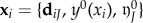, where **d***_iJ_* = [*d_i_*_1_,*d_i_*_2_, … ,*d_i_*_J_] with *y*^1^(**x***_i_*) as the output of interest for training the supervised learner. A standard cross-validation procedure (*k*-fold, *k* = 5) was used to determine the fidelity of the trained supervised model. This procedure was repeated over *s* shuffles (*s* = 10) to demonstrate consistency within model training. Average values of the root mean square error (RMSE) over the shuffles were computed to compare model predictions with the true physics-based solution. The prediction phase then involves determining 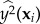 using the trained model and the test data 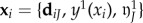, where 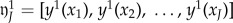. Using the same modelling construct, two other neighbourhood distance metrics ‘mahalanobis’ [42] and ‘cityblock’ were explored and their respective RMSE values evaluated (see the electronic supplementary material for more details).

### 2.4. Gaussian process modelling

A Bayesian approach to GPM was used to train the model for a given set of *l* input vectors 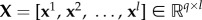, where *q* = 2*J* + 1, with the corresponding output values 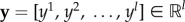 assumed to be available. Using the learned model, output *y*(**x**) can be predicted for a new point **x** [43,44]. The Gaussian process model can be compactly written as2.11
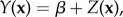
where *β* is an unknown hyperparameter and *Z*(**x**) is a Gaussian stochastic process with zero-mean and covariance2.12
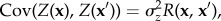
where *R*(**x**,**x**′) is a correlation function that can be tuned to the data and 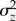 is the process variance. A commonly used choice of correlation function is the stationary family which obeys the *product correlation rule* [45].2.13
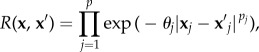
where *θ*_*j*_ ≥ 0 and 0 < *p*_*j*_ ≤ 2 are the hyperparameters. We select *p*_*j*_ = 2 such that the underlying function being modelled is smooth and infinitely differentiable.

In the Bayesian approach to data modelling, two levels of inferences are present. The first level is to infer the parameters given the data and defined using the Bayes' theorem as2.14
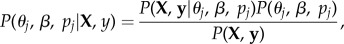
where *P*(*θ*_*j*_,*β,p*_*j*_|**X**,**y**) is the posterior probability of the parameters, *P*(**X**,**y**|*θ*_*j*_,*β*,*p*_*j*_) is the likelihood, *P*(*θ*_*j*_,*β,p*_*j*_) is the prior (assumed to be Gaussian) information about the parameters and *P*(**X**,**y**) is a normalizing constant called the evidence. Note that Gaussian processes are straightforward ways of defining prior distributions for regression and classification problems [46]. Once the hyperparameters are estimated, the second level of inferencing uses these values to estimate *y*(**x**) for a new feature vector **x**. Because of the prior, the observed outputs are realizations of a Gaussian random field, and therefore, we see that the posterior distribution (*P*(**y**(**x**)|**X**,**y**,*θ*_*j*_,*β,p*_*j*_)) of *y*(**x**) is also Gaussian [47], i.e. 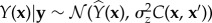. The posterior mean and covariance can be computed as2.15
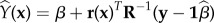
and2.16
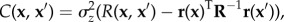
where 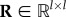 is the correlation matrix computed using the training points; the *ij*th element of this matrix is computed as **R***_ij_* = R(**x***^i^*,**x**^*j*^). 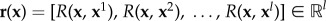 is the correlation between the new point **x** and the training points, and 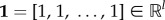 [43,47].

Maximum-likelihood estimation (MLE) was used to compute the hyperparameters ***θ*** = {*θ*_*j*_}, *j* = 1, 2, … , q, *β*, and 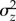 defined in equation (2.12). After dropping the constant terms that do not depend on the hyperparameters, the log-likelihood function becomes2.17
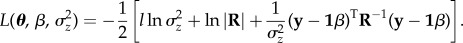


Given the maximum-likelihood estimate of ***θ***, the parameters *β* and 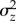 were estimated as2.18
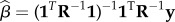
and2.19
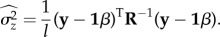
The log-likelihood function can be rewritten using equations (2.17)–(2.19) such that the elements of ***θ*** are the only unknown hyperparameters. These hyperparameters were estimated using the DIRECT global optimization algorithm [48]. Refer to the electronic supplementary material for more details.

## 3. Results

**Figure 3.**
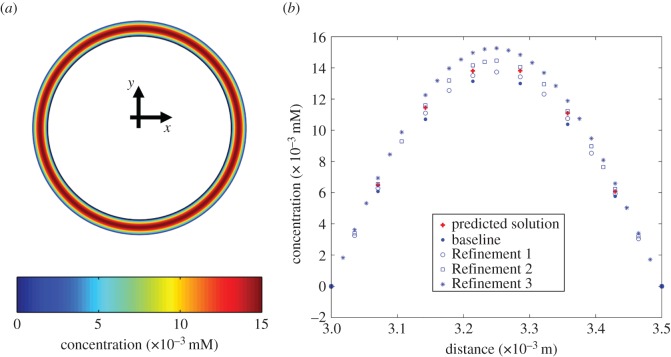
Physics-based solutions for the two-dimensional model of drug-coated balloon delivery and model-based predictions at 1 h after balloon inflation. (*a*) True solution for the bound drug was computed on the mesh setting with the highest density (Refinement 3 in table 1*a*). (*b*) Arterial tissue drug concentration for bound drug for solutions based on four mesh configurations and the model-predicted solution were plotted as a function of cross-sectional depth.

We used GPM to determine whether the data-driven modelling framework can reduce computational time while maintaining accurate solutions to problems of interest. For the case of drug-coated balloon therapy, two-dimensional physics-based models simulating drug transport into the arterial tissue followed by reversible binding predicted arterial distribution patterns of free and bound drug for four mesh configurations (table 1*a*). GPM-based predictions for free and bound arterial drug concentrations qualitatively resembled the true solutions obtained on the two-dimensional time-dependent model with the highest mesh density (figure 3*a* and electronic supplementary material, figure S1a). More specifically, the supervised learner trained using information at the baseline configuration and predicted using Refinement 1 settings generated results that were closer to the true solution (defined by Refinement 3) than the respective true solution at Refinement 2 (figure 3*b* and electronic supplementary material, figure S1b). Note that for these cases, the ‘cityblock’ metric was used to compute 50 nearest neighbour distances for the supervised learner-derived predictions of arterial bound and free drug concentrations, respectively.

### 3.1. Distance metric selection and number of nearest neighbours

**Figure 4.**
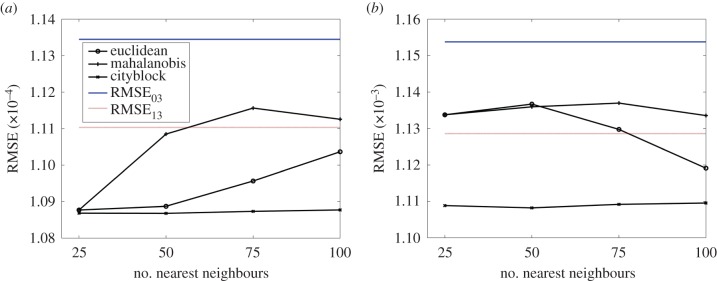
Model accuracy as measured by RMSE varies with distance metric selection and the number of nearest neighbours. RMSE values were computed for three distance metrics and four different values for the number of nearest neighbours. Both (*a*) free and (*b*) bound drug distribution were used as the outputs of interest. RMSE_03_ denotes the RMSE value computed between the solution obtained using the mesh with the lowest density (baseline) and the solution using the mesh with the highest density (Refinement 3). RMSE_13_ denotes the RMSE value computed between the solutions obtained with the Refinements 1 and 3 cases. (Online version in colour.)

The accuracy of models trained using supervised learning demonstrated a strong dependence on the metric used for measuring neighbourhood distances and the number of neighbours used for approximating coarse mesh solutions. In the case of the two-dimensional model, when free tissue drug was considered as the output of interest, RMSE between the true simulation at baseline and Refinement 3 (RMSE_03_) was 1.1345 × 10^−4^ while the RMSE between the true simulation at Refinements 1 and 3 (RMSE_13_) was 1.1104 × 10^−4^ (figure 4*a*). When bound drug was used as the output, RMSE_03_ was 1.2 × 10^−3^ while RMSE_13_ was 1.1 × 10^−3^ (figure 4*b*). These values served as references to validate the performance of the Gaussian process model. Regardless of whether free or bound drug was used as the output, the ‘cityblock’ metric served as the best metric for distance measurement as the corresponding RMSE values computed across any number of nearest neighbours was consistently lower than RMSE_13_. This result indicates that supervised learner-based predictions derived using coarser mesh solutions can serve as more accurate representations of true physics than their computational simulation counterparts, and perhaps closer to the order of the true simulation obtained using meshes with higher density. On the other hand, when metrics such as ‘euclidean’ or ‘mahalanobis’ were used to compute neighbourhood distances, RMSE values of the model were not always lower than RMSE_13_. Note that while the ‘euclidean’ metric is well known in the literature as an ordinary distance between two points (see §2.3), ‘mahalanobis’ distance takes both the distance measurement and the directionality into account, and ‘cityblock’ distance is simply defined using the 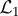 norm. Overall, our findings point to the importance of carefully choosing and validating the distance metric for computing neighbourhood distances as well as the number of nearest neighbours used to compute model performance in order to obtain closer approximations of true physics via the framework of supervised learning on coarse mesh solutions. Using the same modelling construct, several other neighbourhood distance metrics including ‘chebyshev’ [49,50] and ‘minkowski’ and their variations can also be explored.

### 3.2. Extension to memory-intensive simulations

**Figure 5.**
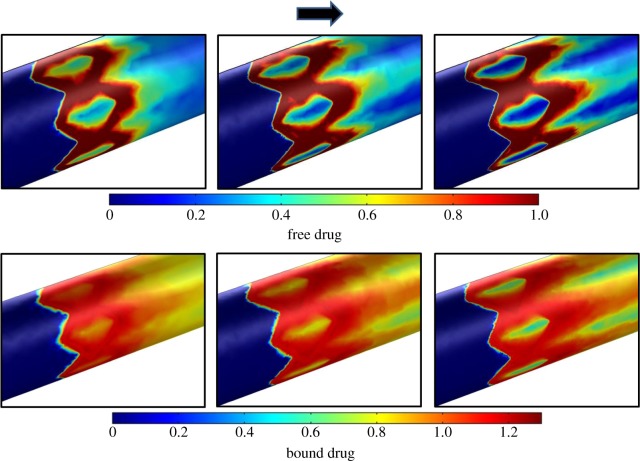
Free and bound arterial drug distribution patterns as predicted by the three-dimensional model of stent-based drug delivery demonstrate qualitative refinement in solution with increased mesh density. The arrow points to the direction of increasing mesh density. Mural drug concentration is normalized to the source and table 1*b* has more details on the three mesh configurations.

**Figure 6.**
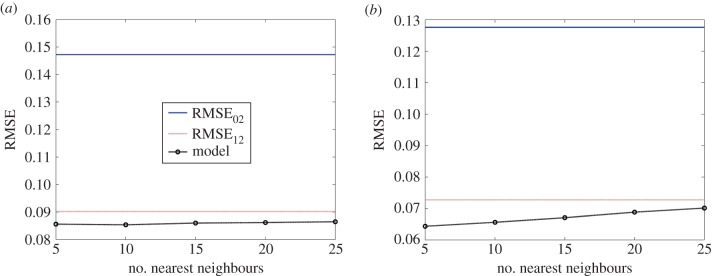
Supervised learner-based predictions for the problem of stent-based drug delivery. Both (*a*) free mural and (*b*) bound tissue drug distribution were used as the outputs of interest. Model accuracy as measured by RMSE is shown to vary with the number of nearest neighbours used to construct the supervised learner. (Online version in colour.)

Having validated the utility of combining the GPM framework to learn from the two-dimensional physiologic simulation data, we extended the framework to physics-based models with increased physiologic and computational complexities (figures 1*b* and 2*b*). Simulations were performed on the three-dimensional model of drug delivery from a stent deployed in an arterial vessel and the effects of steady luminal flow considered as a coupled phenomenon with drug transport and reversible drug binding to arterial tissue (table 1*b*). In contrast to the simulations for the two-dimensional model of drug-coated balloon therapy, the true simulation-based solutions of mural drug distribution for the three-dimensional model of stent-based drug delivery demonstrated an apparent qualitative difference in drug distribution pattern as a function of computational mesh density (figure 5). Also, validation of the supervised learner for the three-dimensional model in the same way as the two-dimensional model was not feasible as our computational resources precluded simulation of the Refinement 3 case (table 1*b*). Therefore, we computed the RMSE values for the supervised learner relative to Refinements 1 and 2 cases, respectively. Models were generated with free and bound drug as outputs using ‘mahalanobis’ metric for nearest neighbour distance measurement and as a function of the number of nearest neighbours (figure 6*a*,*b*). These trends indicate that the model predictions were improved in comparison to that of the true simulation outputs for the Refinement 1 case, but as a function of the number of nearest neighbours and the distance metric.

## 4. Discussion

Computational modelling is being used increasingly in medical device applications, both in the pre-clinical and regulatory domains as well as in emerging clinical applications directly related to patient care. As our ability to characterize patient- and device-specific features in real-time and with micrometre-level precision has improved, such models promise to yield mechanistic and data-driven predictions that will enable personalized care [21]. Yet the computational time required to perform multi-scale high fidelity simulations can be enormous, forcing adoption of high-end computational architecture or reduced fidelity approaches with simplifying assumptions and recognized inaccuracies (e.g. one-dimensional blood flow and lumped parameter models [51], reduced basis methods [52,53], data-driven clinical risk scores [54], etc.). As an alternative, we now demonstrate that use of machine learning on low fidelity, coarse mesh solutions can augment fidelity at a fraction of the computational cost of traditional simulation.

The need to generate high fidelity insight from low fidelity information is not unique to medicine, but rather characteristic of any sufficiently advanced field. Indeed, GPM forms the basis for *Kriging*, an approach that has been widely applied to problems of fidelity optimization in other disciplines including geostatistics as well as aerospace and automobile engineering [43,45,55–57]. Our goal was to leverage this paradigm and demonstrate its utility within a medical context, and in particular that of cardiovascular devices, where the potential for simulation is widely appreciated and accepted, yet its broad reaching impact remains largely anticipated and bottlenecked by modelling inefficiencies. To this effect, by combining nearest neighbour information from low fidelity meshes in a GPM framework, we demonstrate the potential of fused machine learning/physics-based simulation approaches.

### 4.1. Supervised learning for different physics-based models

Using supervised learning, we examined endovascular drug delivery in two contexts, each with unique modelling complexities. In the balloon delivery model, time-dependent simulations predicted intramural free drug diffusion soon after delivery as well as reversible binding of drug and tissue retention after 1 h from the onset of balloon inflation (figure 3*a*). Extraction of data at such long time points naturally extends simulation times. For stent-based delivery, luminal flow transports mural drug from the deployment site to downstream regions in a manner such that pattern of drug imprinted on the mural surface tracks with stent design (figure 5). Here, drugs with low diffusivity in a highly convective environment lead to extremely high Peclet numbers which in turn can result in large instabilities in the numerical solver. Moreover, stent implantation intervenes with the blood flow milieu by introducing perturbations into the boundary layer, causing drug-rich recirculating pools proximal and distal to stent struts [34,36,37,58]. The former aspect can be resolved using highly dense mesh configurations; the latter captured reasonably well with boundary layer meshes. While the two-dimensional model requires high-end computer processors for fast computation due to the model's time-dependent nature, the three-dimensional model is memory intensive and requires large RAM. Each complexity prolongs simulation times, prompting us to explore alternative means by which to obtain fast and accurate solutions to problems of interest.

### 4.2. Role of Gaussian processes

Our approach of combining physics-based computational modelling with data-driven machine learning demonstrates a method to efficiently model complex, multi-scale/multi-physics physiological problems. We specifically selected Gaussian processes because they offer a practical and probabilistic approach to learning and model predictions of real-world problems [32]. However, if this modelling framework is used on an input feature space with several data points and feature vectors, computational time for MLE (equation (2.17)) becomes high. To circumvent part of this problem, we computed the Cholesky decomposition of **R**. This factorization technique produces an upper triangular matrix (**A**) from the diagonal and the upper triangle of the matrix **R**, satisfying the relation **A′A** = **R**, which then allows for efficient matrix inversion. This allowed the posterior mean to be computed using a vector–vector product, i.e. 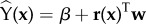, where 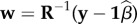. Nevertheless, the combination of physics-based simulations generated on the coarse mesh configurations followed by construction of a supervised learner consumed significantly less time than that was required for the highest mesh density simulation (table 1*a*,*b*). Moreover, time consumed for predicting the output on new nodes using the trained supervised learner is almost negligible. This result highlights the power of harnessing GPM to efficiently approximate data generated from computationally intensive physiologic simulations.

### 4.3. Mesh refinement and nearest neighbour approaches

The concept of increasing mesh density in a successive manner to obtain a solution with better accuracy is core to physics-based computational modelling and mandatory for validating any computational simulation. Yet increased mesh density increases simulation time. Therefore, alternative means by which to obtain fast, more accurate solutions using coarse mesh solutions and without performing high density mesh simulations seems very attractive. We demonstrated that supervised machine learning using Gaussian process modelling on nearest neighbours serves as an appropriate framework to achieve this task.

Nearest neighbour approaches are widely used in the pattern recognition field where one typically solves an optimization problem to identify closest points in a dataset [39,40]. Foundational to such applications is the assumption that some degree of similarity arises purely by virtue of proximity. By assuming that a computational solution space is more or less continuous and void of extreme transitions, we hypothesized that a group comprising solutions of drug concentrations from a node's nearest neighbours carries with it useful information that can facilitate capture of underlying physics more accurately and in the direction of refined mesh solutions. In our study, we observed a strong dependence of the supervised learner's predictive accuracies as a function of the number of nearest neighbours. Interestingly, a higher number of neighbours was found to be optimal for the two-dimensional model (figure 4*a*,*b*) as compared with the three-dimensional setting (figure 6*a*,*b*), likely reflective of a trade-off between added information content and excessive smoothing. In order to implement this framework, one must therefore appreciate the interdependence between the distance metric, number of nearest neighbours and the algorithm for supervised learning. While appropriate combinations can augment efficiency of computationally intensive simulations, inappropriate ones may have an opposite effect.

### 4.4. Study limitations and future directions

We used drug-coated balloon and drug-eluting stent therapies as exemplary medical devices, where modelling solutions require that multiple physics be coupled to gain relevant design and performance insights. Since these device simulations are computationally expensive, we sought alternative means by which to obtain fast and efficient solutions. While we made some assumptions and approximations, such as idealized arterial geometries, these did not detract from the overall scope of the study. Going forward, we hope to apply these learning approaches to examine the impact of pulsatile blood flow [36], other drug, device and procedural settings [35,58–62], disease-induced changes in the arterial wall [63], and patient-specific geometries extracted using image reconstruction to obtain physiologically realistic solutions. As we continue to develop such applications, a number of other approaches can be readily considered. For example, while we present GPM as a useful learning framework, other supervised learning techniques such as artificial neural networks and support vector machines could be implemented in a similar fashion, serving as efficient approximations to standard physics-based simulations [39,40,64,65]. Furthermore, several other methods related to fidelity optimization and applications to data mining exist within the literature [66–69], and could be leveraged in conjunction with the presented framework. Also, when standard linear dimensionality reduction techniques such as principal component analysis (PCA) and nonlinear dimensionality reduction techniques such as kernel PCA are used to pre-condition the input feature space prior to supervised learning [70], computational time for model training can be further reduced. Pseudocodes are provided in the electronic supplementary material to assist those interested to implement our supervised learning schema for specific applications.

## 5. Conclusion

Emerging applications in medicine and medical device design require that we find ways to provide high fidelity insight efficiently. Fusion of machine learning with standard physics-based computational models is perfectly suited to meet this challenge. In the context of endovascular device-based drug therapies, we demonstrate the potential of fidelity augmentation, wherein high fidelity inferences were drawn quickly from low fidelity information. Use of such a framework may help computational tools overcome limiting bottlenecks, allowing them to be realized in real-world medical applications characterized by inherent modelling complexities with a time critical nature.

## Supplementary Material

Supplemental Information
