# Supplementary material for: Enhancing physiologic simulations using supervised learning on coarse mesh solutions
Source: J R Soc Interface. 2015 Mar 6;12(104):20141073. doi: 10.1098/rsif.2014.1073 (PMC4345474; doi:10.1098/rsif.2014.1073)
Supplement: Supplemental Information [file rsif20141073supp1.pdf]

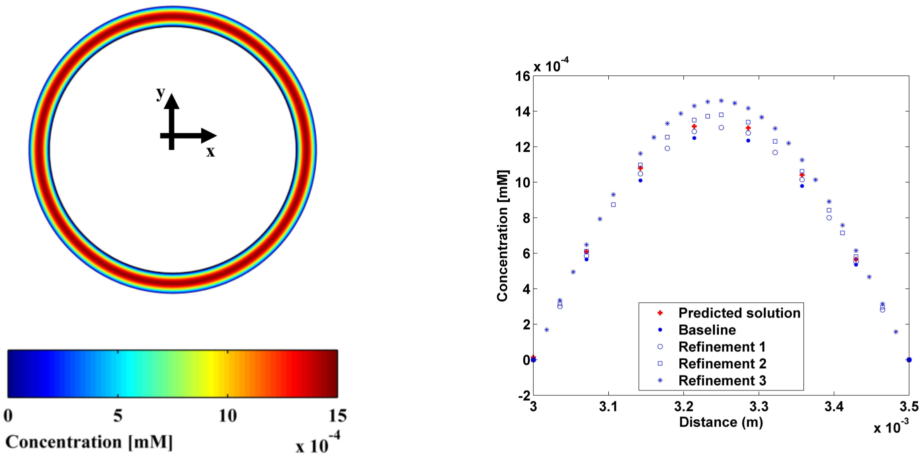

(a) (b)

Figure S1: (S1a) Physics-based solution for the 2D model of drug-coated balloon delivery at 1 hour after balloon inflation. Note that true solution for the free drug computed on the mesh setting with the highest density is shown. (S1b) Comparison of physics-based solutions for the 2D model of drug-coated balloon delivery with model-based predictions at 1 hour after balloon inflation. Arterial tissue drug concentration for free drug for solutions based on four mesh configurations and the model-predicted solution were plotted as a function of depth.

Supplemental information

Distance metrics

The 'mahalanobis' distance metric is a multi-dimensional generalization of the idea of measuring how many standard deviations away a point is from a distribution that it belongs. It takes into account the covariance among the variables in calculating distances. For example, if $\mathbf{x}^i$ and $\mathbf{x}^j$ are two points from the same distribution which has covariance matrix $C(\mathbf{x}^i, \mathbf{x}^j)$, then the Mahalanobis distance is given by $\left[(\mathbf{x}^i - \mathbf{x}^j)' C^{-1} (\mathbf{x}^i - \mathbf{x}^j)\right]^{\frac{1}{2}}$. In the limiting case where the covariance matrix is an identity matrix, the 'mahalanobis' distance reduces to the Euclidean distance.

For two vectors $\mathbf{x}^i, \mathbf{x}^j$ in a p-dimensional real vector space, the 'cityblock' metric is the sum of the lengths of the projections of the line segment between the points onto the coordinate axes. Taxicab geometry and Manhattan distance are two other names used to describe 'cityblock' metric as they allude to the shortest path a cab could take between two intersections within the borough of Manhattan, New York.

Algorithm for supervised learning on coarsed mesh solutions

In what follows, we outline a set of pseudocodes for supervised learning of the data generated from coarse mesh solutions. While our approach used Gaussian process modeling for supervised learning, several other methods can be used through substitution of **Algorithm 5**. Similarly, we used three metrics to compute nearest neighbor distances (‘euclidean’, ‘cityblock’ and ‘mahalanobis’); other metrics such as ‘minkowski’ and ‘chebyshev’ could be considered in **Algorithms 3** & **4**.

Algorithm 1 Supervised learning on coarsed mesh solutions

Run **Algorithm** 2
for $s := 1$ to $maxShuffles$ **do**
    for $i := 1$ to $maxNodes$ **do**
        Run **Algorithm** 3 on a single mesh node i
        XTRAIN(i,:) $\leftarrow$ **xTrain**$_i$
        yTrain(i) $\leftarrow yTrain_i$
        Run **Algorithm** 4 on a single mesh node i
        XTEST(i,:) $\leftarrow$ **xTest**$_i$
        yTest(i) $\leftarrow yTest_i$
    end for
    Run **Algorithms** 5, 6 & 7
end for
Compute mean RMSE averaged over $maxShuffles$

Algorithm 2 Import mesh and drug distribution data

1: **function** IMPORTDATA ▷ Import data for refinements 0 (**R0**), 1 (**R1**), 2 (**R2**) & 3 (**R3**)
2:     **X0** $\leftarrow$ Mesh coordinates of **R0**
3:     **y0** $\leftarrow$ Solution at **R0**
4:     **indx1** $\leftarrow$ Index position of nodes in **R1**, where coordinates of **R1** coincide with **R0**
5:     **X1** $\leftarrow$ Mesh coordinates of **R1** at nodes defined by **indx1**
6:     **y1** $\leftarrow$ Solution at **R1** at nodes defined by **indx1**
7:     **repeat** Steps 4 - 6
8:     **until R2** & **R3**
9: **end function**

Algorithm 3 Data for model training

function GETTRAININGDATA ▷ Get training data from mesh refinements 0 & 1
  $\mathbf{x}_i \leftarrow$ Coordinate of node of interest in $\mathbf{X0}$
  $y_i^0 \leftarrow$ Solution at node of interest in $\mathbf{y0}$
  $\mathbf{d}_{iJ} \leftarrow$ Distances of J nearest neighbor nodes from $\mathbf{x}_i$
  $\mathfrak{y}_J^0 \leftarrow$ Solution at J nearest neighbor nodes in $\mathbf{y0}$
  $\mathbf{xTrain}_i \leftarrow \{\mathbf{d}_{iJ}, y_i^0, \mathfrak{y}_J^0\}$
  $yTrain_i \leftarrow$ Solution at node of interest in $\mathbf{y1}$
end function

Algorithm 4 Data for model testing

function GETTESTINGDATA ▷ Get testing data from mesh refinements 1 & 2
  $\mathbf{x}_i \leftarrow$ Coordinate of node of interest in $\mathbf{X1}$
  $y_i^1 \leftarrow$ Solution at node of interest in $\mathbf{y1}$
  $\mathbf{d}_{iJ} \leftarrow$ Distances of J nearest neighbor nodes from $\mathbf{x}_i$
  $\mathfrak{y}_J^1 \leftarrow$ Solution at J nearest neighbor nodes in $\mathbf{y1}$
  $\mathbf{xTest}_i \leftarrow \{\mathbf{d}_{iJ}, y_i^1, \mathfrak{y}_J^1\}$
  $yTest_i \leftarrow$ Solution at node of interest in $\mathbf{y2}$
end function

Algorithm 5 Perform model training

function MODELTRAINING ▷ Use Gaussian process modeling to estimate hyperparameters
  Initial guess on hyperparameters
  $i \leftarrow 1$
  while $i \leq maxIter \parallel i \leq maxEvals$ **do**
    Compute correlation matrix (Equation 13)
    For given $\boldsymbol{\theta}$, compute $\widehat{\beta}$ and $\widehat{\sigma_z^2}$ (Equations 18 & 19) and substitute in Equation 17
    Perform maximum likelihood estimation of $\boldsymbol{\theta}$
    $i \leftarrow i + 1$
  end while
end function

Algorithm 6 Perform model testing

function MODELPREDICTIONS ▷ Use trained model to predict on test data
  $\widehat{\mathbf{y}} \leftarrow$ Predict on **XTEST** given the estimated hyperparameters
end function

Algorithm 7 Error calculations

function RMSECOMPUTATION ▷ Compute root mean square error

  Initialize $error_{03}$, $error_{13}$, $error_{23}$, $errorModel$

  for i:=1 to size($\mathbf{y0}$,1) **do**

    $error_{03} \leftarrow error_{03} + (\mathbf{y0}(i) - \mathbf{y3}(i))^2$

    $error_{13} \leftarrow error_{13} + (\mathbf{y1}(i) - \mathbf{y3}(i))^2$

    $error_{23} \leftarrow error_{23} + (\mathbf{y2}(i) - \mathbf{y3}(i))^2$

    $errorModel \leftarrow errorModel + (\widehat{\mathbf{y}}(i) - \mathbf{y3}(i))^2$

  end for

  $RMSE_{03} \leftarrow sqrt(error_{03}/size(\mathbf{y0}, 1))$

  $RMSE_{13} \leftarrow sqrt(error_{13}/size(\mathbf{y0}, 1))$

  $RMSE_{23} \leftarrow sqrt(error_{23}/size(\mathbf{y0}, 1))$

  $RMSEModel \leftarrow sqrt(errorModel/size(\mathbf{y0}, 1))$

end function

Media summary

Medical applications are beginning to embrace the potential of computational models that promise insights into device function and patient-specific performance. Yet the explosion of knowledge and medical data is outstripping processing abilities, particularly in real time decision-support. Fusion of machine learning with standard physics-based computational models offers a way to meet these emerging challenges. In the context of cardiovascular devices, we demonstrate a framework that permits high accuracy inferences to be drawn quickly from low accuracy information at a fraction of the computational cost. Such tools can help overcome processing bottlenecks, facilitating realization of computing potential in time-critical medical applications.
